# Prior COVID-19 Infection, Mental Health, Food and Financial Insecurity, and Association With COVID-19 Vaccination Coverage and Intent Among College-Aged Young Adults, US, 2021

**DOI:** 10.5888/pcd18.210260

**Published:** 2021-12-16

**Authors:** Kimberly H. Nguyen, Shannon Irvine, Rebecca Epstein, Jennifer D. Allen, Laura Corlin

**Affiliations:** 1 Department of Public Health & Community Medicine, Tufts University School of Medicine, Boston, Massachusetts; 2 Department of Community Health, Tufts University, Medford, Massachusetts; 3 Department of Civil and Environmental Engineering, Tufts University School of Engineering, Medford, Massachusetts

## Abstract

**Introduction:**

More than 700,000 COVID-19 cases have been linked to American colleges and universities since the beginning of the pandemic. However, studies are limited on the effects of the pandemic on college-aged young adults and its association with their COVID-19 vaccination status and intent.

**Methods:**

Using the Census Bureau’s Household Pulse Survey (HPS), a large, nationally representative survey fielded from April 14 through May 24, 2021, we assessed the effects of the pandemic (COVID-19 infection, mental health, food and financial security) on COVID-19 vaccination coverage (≥1 dose) and intentions toward vaccination among college-aged young adults in the United States (N = 6,758). We examined factors associated with vaccination coverage and intent, and reasons for not getting vaccinated.

**Results:**

Approximately one-fifth (19.6%) of college-aged young adults had a previous diagnosis of COVID-19, 43.5% and 39.1% reported having anxiety or depression, respectively, 10.9% reported that they sometimes or often did not have enough food to eat, and 22.6% and 12.3% found it somewhat or very difficult, respectively, to pay for household expenses. Of college-aged young adults, 63.1% had received at least 1 dose of the COVID-19 vaccine, 15.4% probably would be vaccinated or were unsure about getting the vaccine, and 14.0% probably will not or definitely will not get vaccinated. Adults who were non-Hispanic Black (vs non-Hispanic White) or had food or financial insecurities (vs did not) were less likely to be vaccinated or intend to be vaccinated. Among adults who probably will not or definitely will not be vaccinated, more than one-third said that they did not believe a vaccine was needed.

**Conclusion:**

Ensuring high and equitable vaccination coverage among college-aged young adults is critical for safely reopening in-person learning and resuming prepandemic activities.

**Summary**

**What is already known on this topic?**

Since mid-2020, young adults have had the highest prevalence of COVID-19 cases and are least likely to be vaccinated or to intend to be vaccinated for COVID-19.

**What is added by this report?**

Approximately 63% of college-aged young adults had received at least 1 dose of the COVID-19 vaccine, 15.4% probably will or were unsure about getting the vaccine, and 14.0% probably will not or definitely will not get vaccinated. Students who were non-Hispanic Black or had food or financial insecurities were less likely than their counterparts to be vaccinated.

**What are the implications for public health practice?**

Ensuring high and equitable vaccination coverage is critical for safely reopening in-person learning as well as resuming prepandemic activities.

## Introduction

The COVID-19 pandemic has resulted in disruptions to the lives and education of college students (1,2). Because of college lockdowns, cancellation of classes, social distancing recommendations, and shifts in instructional methods, most college students have experienced changes to their daily lives, including quarantining or living at home, having mental health issues, experiencing housing and food insecurity, and having financial hardships (3–9). A recent survey found that more than 700,000 COVID-19 cases have been linked to American colleges and universities since the beginning of the pandemic (10). Since mid-2020, the highest prevalence of COVID-19 cases has been among young adults (adults aged 18–24, followed by those aged 25–34) (11,12). Young adults feel less at risk than older adults for the consequences of COVID-19 and are less likely to adhere to strategies to minimize risk of exposure to COVID-19, such as mask wearing or social distancing (13). Because young adults are less likely to adhere to mitigation strategies, they are more likely to spread the SARS-CoV-2 virus to others and are at risk of COVID-19–related morbidity and mortality (12,13).

Although the COVID-19 vaccine is available now to everyone 12 years or older, young adults have been less likely than older adults to get vaccinated or intend to get vaccinated (14,15). Vaccination administration data reported to the Centers for Disease Control and Prevention (CDC) from December 14, 2020, to May 22, 2021, indicated that young adults (adults aged 18–29 and 30–49) had lower vaccination rates (38% and 49%, respectively) than adults aged 65 or older (79%) (14). Furthermore, a national survey found that 23% of adults aged 18 to 39 reported that they would probably get or were unsure about getting a COVID-19 vaccine, and 24% reported that they would probably not or would definitely not get vaccinated (15). Studies found that although college students were more likely to get a COVID-19 vaccine than an influenza vaccine, students perceived that other young adults would be less likely to be vaccinated for COVID-19 and would not think vaccination was as important (16,17). These studies suggest that developing and testing norms-based intervention strategies, such as personalized normative feedback, would increase COVID-19 vaccination among college students.

We used the Health Belief Model to conceptualize mental health status, food and financial insecurity, and sociodemographic factors as barriers to vaccination, and prior COVID-19 infection as related to perceived risk in a model to understand factors associated with COVID-19 vaccination and intent (18). Understanding barriers and perceived risk among college-age students and their association with COVID-19 vaccination status and intent is needed to tailor messages to increase vaccine uptake in this population. The objective of this study was to examine state and national estimates of, and factors associated with, COVID-19 vaccination coverage and intent. Furthermore, we assessed reasons for not vaccinating among a large, national sample of college-aged young adults.

## Methods

This study used data from the Household Pulse Survey (HPS), a large, nationally representative household survey conducted by the US Census Bureau since April 2020 to help understand household experiences during the COVID-19 pandemic. The design of this survey is described elsewhere (19–21). The survey is fielded once or twice per month with approximately 75,000 respondents during each wave. We combined data from 3 survey waves (April 14–April 26, April 28–May 10, and May 12–24, 2021) for this study; response rates ranged from 6.6% to 7.4% (22). This study was reviewed by the Tufts University Health Sciences Institutional Review Board and was considered not to be human subjects research.

### College-aged young adults

Respondents were asked “What is the highest degree or level of school you have completed?” and “How many members of your household, including yourself, are currently taking, or were planning to take classes this term from a college, university, community college, trade school, or other occupational school (such as a cosmetology school or a school of culinary arts)?” Respondents who answered “some college, but degree not received or is in progress” to the former question and one or more to the latter question were categorized as college-aged young adults to reduce the potential for misclassification if they were not in fact college students. To further reduce potential for misclassification (ie, if a respondent had some college education, but was not currently in college and had someone else in the household, such as children, in college), the analysis was restricted to respondents aged 39 or younger (n = 6,803).

### COVID-19 questions

The HPS asks questions on COVID-19 diagnosis and vaccination coverage, intent, and reasons for not vaccinating. COVID-19 diagnosis was assessed by the following question: “Has a doctor or other health care provider ever told you that you have COVID-19?” (yes/no/not sure). COVID-19 vaccination receipt (≥1 dose) was assessed with the following question: “Have you received a COVID-19 vaccine?” (yes/no). People who did not answer the question about whether they had been vaccinated were excluded from the analysis (n = 45). Among unvaccinated adults, intent to be vaccinated was assessed with the following question: “Once a vaccine to prevent COVID-19 is available to you, would you . . . definitely, probably, be unsure about, probably not, or definitely not get(ting) a vaccine.” Because the vaccination intent questions were asked only of those who were not vaccinated, assessing intent over time would show bias as more people got vaccinated (reducing the sample size of those who are asked about intent). To reduce this potential for bias, the denominator for vaccination intent was everyone in the sample, including those who were vaccinated. Unvaccinated respondents who did not definitely plan to be vaccinated were categorized as 1) probably will get vaccinated/unsure about getting vaccinated and 2) probably will not/definitely will not get vaccinated.

Among nonvaccinated individuals who definitely did not intend to get vaccinated, respondents were asked reasons for not getting vaccinated: “Which of the following, if any, are reasons that you [probably will/be unsure about/probably won’t/definitely won’t] get a COVID-19 vaccine?” Response options, in which they could select all that apply, were: 1) I am concerned about possible side effects of a COVID-19 vaccine, 2) I don’t know if a COVID-19 vaccine will work, 3) I don’t believe I need a COVID-19 vaccine, 4) I don’t like vaccines, 5) My doctor has not recommended it, 6) I plan to wait and see if it is safe and may get it later, 7) I think other people need it more than I do right now, 8) I am concerned about the cost of a COVID-19 vaccine, 9) I don’t trust COVID-19 vaccines, 10) I don’t trust the government, and 11) other. These questions underwent expert review at the US Census Bureau and federal partner agencies, as well as cognitive testing in laboratories at CDC’s National Center for Health Statistics (20).

### Sociodemographic variables

Sociodemographic variables assessed were age group (18–24, 25–29, 30–34, and 35–39 y), sex (female, male), and race and ethnicity (Hispanic, non-Hispanic Asian, non-Hispanic Black, non-Hispanic other/multiple races, and non-Hispanic White).

#### Food and financial security

Food sufficiency was assessed through the following question: “Getting enough food can also be a problem for some people. In the last 7 days, which of these statements best describes the food eaten in your household?” This variable was categorized as 1) having enough food to eat in the past 7 days, regardless of whether the food is always the kind of food that the respondent wanted to eat, and 2) sometimes or often not having enough to eat. Financial security was assessed through the following question: “In the last 7 days, how difficult has it been for your household to pay for usual household expenses, including but not limited to food, rent or mortgage, car payments, medical expenses, student loans, and so on?” Responses were categorized as “very,” “somewhat,” “a little,” and “not at all.”

#### Mental health questions

Questions about anxiety and depression were modified from a validated 2-item Patient Health Questionnaire–2 (PHQ–2) and the 2-item Generalized Anxiety Disorder (GAD–2) scale (23,24) (Box). Responses to both scales were assigned a numerical value ranging from 0 to 3 (not at all = 0, several days = 1, more than half the days = 2, and nearly every day = 3). Scores from each scale were summed; a score of ≥3 on the PHQ–2 was categorized as symptoms of depression (hereinafter referred to as depression), and a sum of ≥3 on the GAD–2 was categorized as symptoms of anxiety (hereinafter referred to as anxiety).

**Box. Questions Adapted from Patient Health Questionnaire–2 and the Generalized Anxiety Disorder Scale (23,24)**

**Patient Health Questionnaire–2**

1) “Over the last 7 days, how often have you been bothered by . . . having little interest or pleasure in doing things? Would you say not at all, several days, more than half the days, or nearly every day?”

2) “Over the last 7 days, how often have you been bothered by . . . feeling down, depressed, or hopeless? Would you say not at all, several days, more than half the days, or nearly every day?”

**Generalized Anxiety Disorder–2 Scale**

1) “Over the last 7 days, how often have you been bothered by the following problems . . . Feeling nervous, anxious, or on edge? Would you say not at all, several days, more than half the days, or nearly every day?”

2) “Over the last 7 days, how often have you been bothered by the following problems . . . Not being able to stop or control worrying? Would you say not at all, several days, more than half the days, or nearly every day?

### Analysis

Prevalence of COVID-19 diagnosis, food and financial insecurity, and mental health conditions were assessed overall and by vaccination coverage (≥1 dose) and intent to be vaccinated (probably will get vaccinated/unsure about getting vaccinated or probably will not/definitely will not) among college-aged young adults aged 39 or younger. We conducted multivariable regression models to examine factors associated with at least 1 dose of COVID-19 vaccine receipt and intent. We examined proportions and 95% CIs for reasons for not getting vaccinated among adults who were probably or unsure about getting vaccinated and probably will not or definitely will not be vaccinated. Contrast tests for the differences in proportions, comparing each category to the referent category, were conducted with a .05 significance level (α = .05). Analyses accounted for the survey design and weights to ensure the sample was representative of the US population by using Stata version 16.1 (StataCorp LLC).

## Results

Approximately one-half (47.4%) of adults in this sample were aged 18 to 24, 20.2% were aged 25 to 29, 18.2% were aged 30 to 34, and 14.2% were aged 35 to 39 years. By race and ethnicity, 54.8% were non-Hispanic White, 23.2% were Hispanic, 12.1% were non-Hispanic Black, 5.2% were non-Hispanic Asian, and 4.7% were non-Hispanic other/multiple races (Table 1). Moreover, 10.9% reported that they sometimes or often did not have enough food to eat in the past 7 days, and 22.6% and 12.3% reported that it was somewhat or very difficult, respectively, to pay for household expenses in the past 7 days. More than one-third reported having anxiety (43.5%) or depression (39.1%) in the past 7 days, and 19.6% reported a previous diagnosis of COVID-19.

**Table 1:** Characteristics of College Students, Household Pulse Survey, US, April 14–May 24, 2021^a^

Characteristic	Overall		Previous COVID-19 Diagnosis, % (95% CI)
	Unweighted n	% (95% CI)	
**All adults aged 18–39**	6,758	100	19.6 (17.9–21.5)
**Age group, y**			
18–24	2,327	47.4 (45.7–49.2)	20.9 (18.5–23.6)
25–29	1,277	20.2 (18.7–21.8)	21.0 (17.5–25.0)
30–34	1,505	18.2 (16.9–19.6)	16.4 (13.5–19.6)
35–39	1,649	14.2 (13.2–15.2)	17.6 (14.8–20.7)
**Sex**			
Male	2,670	47.5 (46.3–48.6)	18.9 (16.3–21.7)
Female	4,088	52.5 (51.4–53.7)	20.3 (18.4–22.4)
**Race and ethnicity**			
Hispanic	1,269	23.2 (22.0–24.5)	25.8 (21.8–30.2)
Non-Hispanic Asian	334	5.2 (4.6–6.0)	13.3 (8.5–20.4)
Non-Hispanic Black	748	12.1 (11.0–13.2)	16.7 (13.4–20.8)
Non-Hispanic other/multiple races	427	4.7 (4.2–5.3)	12.8 (9.1–17.7)
Non-Hispanic White	3,980	54.8 (53.4–56.1)	18.9 (16.7–21.3)
**Food sufficiency**			
Sometimes/often not enough to eat	592	10.9 (9.6–12.4)	19.9 (14.6–26.6)
Enough food to eat	4,136	89.1 (87.6–90.4)	18.2 (16.4–20.3)
**Difficulty paying for household expenses**			
Not at all difficult	1,982	34.8 (32.8–36.9)	17.0 (14.7–19.6)
A little difficult	1,689	30.3 (28.4–32.3)	18.3 (15.5–21.6)
Somewhat difficult	1,314	22.6 (20.9–24.4)	19.5 (15.9–23.6)
Very difficult	882	12.3 (11.1–13.6)	21.8 (17.7–26.6)
**Anxiety**			
Yes	1,705	43.5 (41.1–45.9)	19.2 (16.0–23.0)
No	2,155	56.5 (54.1–58.9)	17.8 (15.1–20.8)
**Depression**			
Yes	1,444	39.1 (36.7–41.5)	19.2 (15.6–23.3)
No	2,416	60.9 (58.5–63.3)	18.0 (15.5–20.7)

a All estimates were weighted to be representative of the US population.

**Figure 1:**
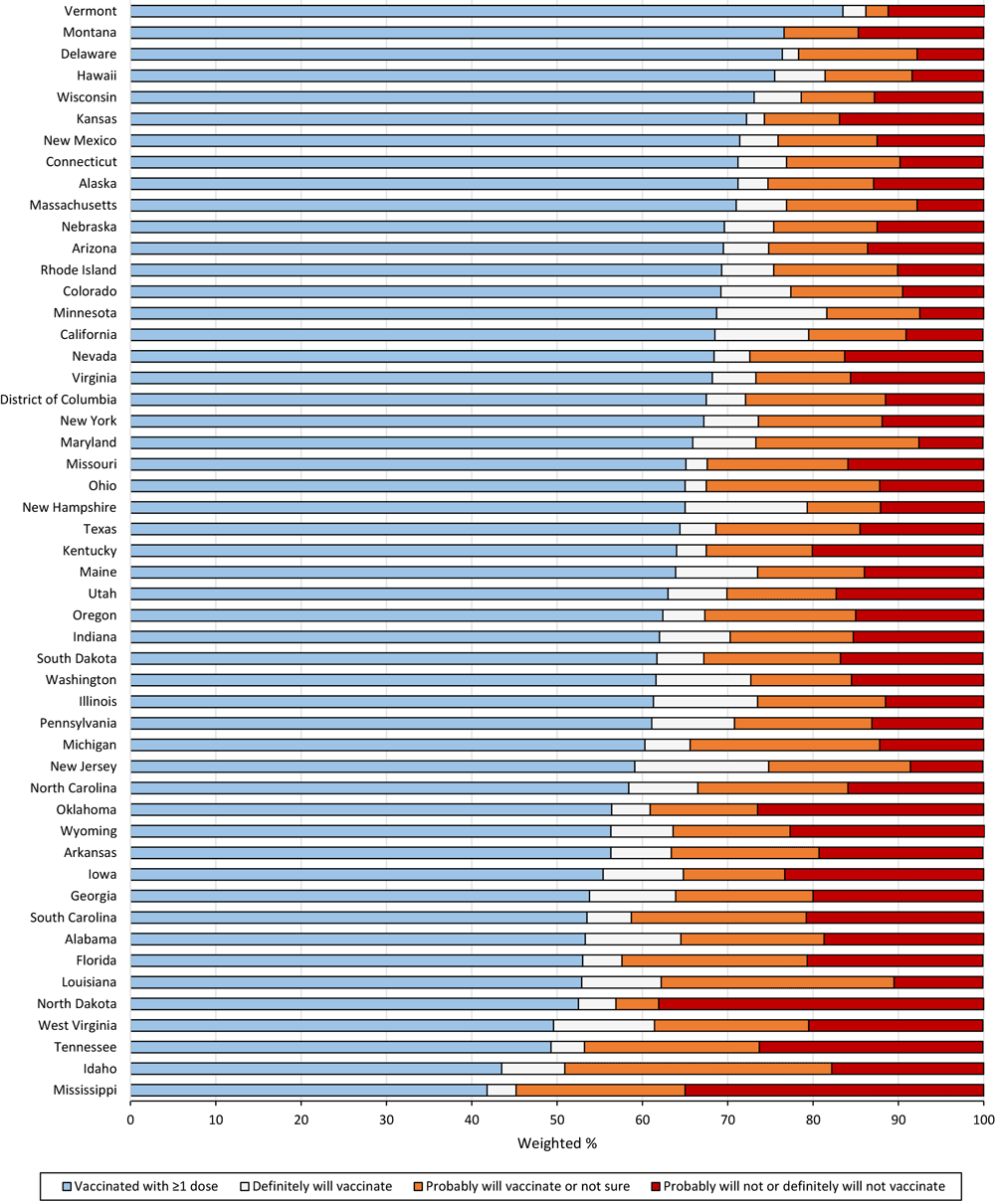
COVID-19 vaccination coverage and intention to vaccinate among college students by state, US, Household Pulse Survey, April 14–May 24, 2021.

**Figure 2:**
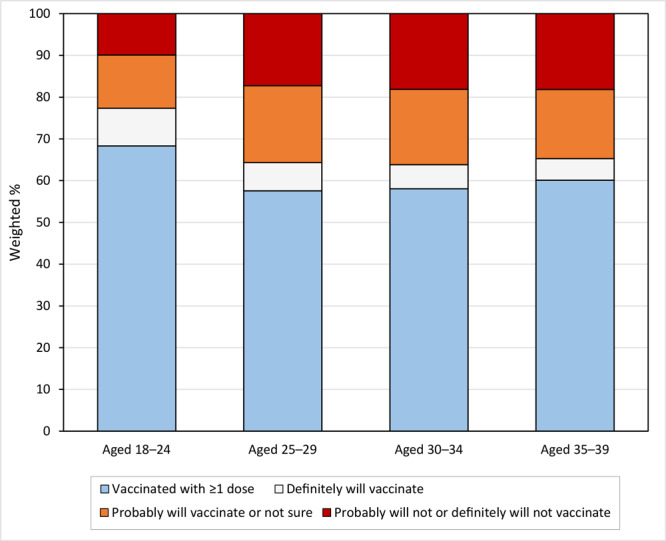
COVID-19 vaccination coverage and intention to vaccinate among college students by age group, US, Household Pulse Survey, April 14 –May 24, 2021.

Approximately 63% of college-aged young adults in this sample had received at least 1 dose of the COVID-19 vaccine, 15.4% reported that they probably would be vaccinated or were unsure about getting the vaccine, and 14.0% reported that they probably will not or definitely will not get vaccinated (Table 2). Vaccination coverage ranged from 42.8% in Mississippi to 83.5% in Vermont (Figure 1). By age, vaccination coverage was higher among adults aged 18 to 24 (68.3%) than among adults aged 25 to 29 (57.6%), 30 to 34 (58.1%), and 35 to 39 (60.1%) (Table 2 and Figure 2).

**Table 2:** Prevalence of and Factors Associated With COVID-19 Vaccination (≥1 Dose) and Intent to Get Vaccinated, Household Pulse Survey, US, April 14–May 24, 2021

Characteristic	COVID-19 Vaccination (≥1 Dose)		COVID-19 Vaccination Intent			
			Probably Will/Unsure About Getting the Vaccine		Definitely or Probably Will Not Get the Vaccine	
	% (95% CI)	aPR (95% CI)^a^	% (95% CI)	aPR (95% CI)^a^	% (95% CI)	aPR (95% CI)^a^
**All**	63.1 (60.9–65.3)	** —**	15.4 (13.9–17.0)	** —**	14.0 (12.9–15.3)	** —**
**Age group, y**						
18–24	68.3 (65.0–71.5)	1 [Reference]	12.8 (11.0–14.7)	1 [Reference]	9.9 (8.4–11.7)	1 [Reference]
25–29	57.6 (53.2–61.8)^b^	0.93 (0.84–1.03)	18.4 (14.8–22.8)^b^	1.06 (0.77–1.46)	17.2 (14.2–20.8)^b^	1.71 (1.21–2.42)
30–34	58.1 (54.2–61.9)^b^	0.94 (0.86–1.03)	18.0 (15.7–20.7)^b^	1.16 (0.87–1.54)	18.1 (15.3–21.4)^b^	1.61 (1.17–2.22)
35–39	60.1 (56.2–63.9)^b^	0.93 (0.84–1.03)	16.6 (14.2–19.3)^b^	1.24 (0.97–1.59)	18.2 (15.7–20.9)^b^	1.45 (1.04–2.02)
**Sex**						
Male	63.1 (59.6–66.5)	1.00 (0.93–1.07)	16.1 (13.7–18.8)	1.10 (0.88–1.38)	13.2 (11.5–15.1)	0.91 (0.73–1.14)
Female	63.1 (60.8–65.4)	1 [Reference]	14.8 (13.1–16.8)	1 [Reference]	14.8 (13.3–16.5)	1 [Reference]
**Race and ethnicity**						
Hispanic	66.9 (62.2–71.3)	1.05 (0.96–1.15)	16.8 (13.6–20.6)	1.17 (0.83–1.66)	8.1 (6.3–10.3)^b^	0.57 (0.40–0.82)
Non-Hispanic Asian	84.4 (79.6–88.3)^b^	1.33 (1.22–1.44)	4.6 (2.7–7.7)^b^	0.38 (0.17–0.83)	—c	0.04 (0.004–0.29)
Non-Hispanic Black	42.7 (36.8–48.8)^b^	0.69 (0.59–0.82)	27.0 (22.0–32.3)^b^	1.95 (1.44–2.63)	20.6 (17.0–24.8)	0.97 (0.75–1.25)
Non-Hispanic other/multiple races	65.0 (58.2–71.1)	1.06 (0.94–1.21)	12.8 (8.9–18.2)	0.79 (0.49–1.27)	13.3 (9.5–18.3)	0.62 (0.34–1.11)
Non-Hispanic White	63.8 (61.3–66.3)	1 [Reference]	13.5 (11.9–15.3)	1 [Reference]	16.4 (14.7–18.2)	1 [Reference]
**Food sufficiency**						
Sometimes/often not enough to eat	46.2 (38.8–53.7)^b^	0.83 (0.70–0.98)	23.8 (18.5–30.1)^b^	1.40 (1.00–1.96)	24.2 (18.9–30.6)^b^	1.34 (0.98–1.82)
Enough food to eat	65.5 (62.9–68.1)	1 [Reference]	13.9 (12.2–15.7)	1 [Reference]	12.8 (11.5–14.1)	1 [Reference]
**Difficulty paying for household expenses**						
Not at all difficult	70.8 (67.0–74.2)	1 [Reference]	12.0 (9.9–14.6)	1 [Reference]	10.1 (8.2–12.4)	1 [Reference]
A little difficult	66.1 (62.6–69.4)^b^	0.94 (0.87–1.01)	13.7 (11.2–16.7)	1.20 (0.90–1.60)	11.9 (10.1–14.1)	1.16 (0.84–1.62)
Somewhat difficult	61.6 (57.4–65.6)^b^	0.93 (0.83–1.04)	18.4 (15.4–21.7)^b^	1.30 (0.97–1.73)	14.2 (11.5–17.4)^b^	1.45 (0.92–2.28)
Very difficult	45.9 (40.4–51.5)^b^	0.71 (0.62–0.82)	21.4 (17.4–26.1)^b^	1.51 (1.09–2.09)	25.1 (20.4–30.4)^b^	2.24 (1.53–3.27)
**Anxiety**						
Yes	63.2 (59.5–66.7)	1.06 (0.97–1.16)	15.3 (12.4–18.8)	0.97 (0.72–1.31)	13.2 (11.2–15.5)	0.78 (0.61–1.00)
No	64.9 (61.6–68.1)	1 [Reference]	14.4 (12.4–16.6)	1 [Reference]	14.1 (12.3–16.2)	1 [Reference]
**Depression**						
Yes	62.7 (58.5–66.7)	0.99 (0.91–1.08)	15.1 (11.9–18.9)	0.93 (0.69–1.25)	12.4 (10.2–15.0)	0.77 (0.58–1.02)
No	65.1 (62.0–68.1)	1 [Reference]	14.6 (12.5–17.0)	1 [Reference]	14.5 (12.6–16.6)	1 [Reference]
**Previous COVID-19 diagnosis**						
Yes	56.1 (50.9–61.2)^b^	0.91 (0.81–1.01)	20.6 (17.3–24.3)^b^	1.49 (1.14–1.94)	15.0 (12.1–18.4)	0.88 (0.69–1.14)
No	65.1 (62.8–67.3)	1 [Reference]	13.8 (12.3–15.5)	1 [Reference]	13.9 (12.7–15.2)	1 [Reference]

Abbreviation: aPR, adjusted prevalence ratio.

a aPR adjusted for age, sex, race or ethnicity, previous COVID-19 diagnosis, anxiety or depressive status, current food sufficiency, and difficulty paying for household expenses. All proportions and adjusted prevalence ratios were weighted to be representative of the US population.

b Significant difference (*P* < .05) in proportions between group specified and referent group; determined by contrast tests for proportions.

c Estimates were suppressed because of relative standard error >30%.

**Table:** 

State	Vaccinated With 1 or More Doses, %	Definitely Will Vaccinate, %	Probably Will Vaccinate or Not Sure, %	Probably Will Not or Definitely Will Not Vaccinate, %
Mississippi	42	3	20	35
Idaho	44	7	31	18
Tennessee	49	4	21	26
West Virginia	50	12	18	20
North Dakota	52	4	5	38
Louisiana	53	9	27	10
Florida	53	5	22	21
Alabama	53	11	17	19
South Carolina	54	5	21	21
Georgia	54	10	16	20
Iowa	55	9	12	23
Arkansas	56	7	17	19
Wyoming	56	7	14	23
Oklahoma	56	5	13	26
North Carolina	58	8	18	16
New Jersey	59	16	17	9
Michigan	60	5	22	12
Pennsylvania	61	10	16	13
Illinois	61	12	15	12
Washington	62	11	12	16
South Dakota	62	6	16	17
Indiana	62	8	14	15
Oregon	62	5	18	15
Utah	63	7	13	17
Maine	64	10	13	14
Kentucky	64	4	12	20
Texas	64	4	17	15
New Hampshire	65	14	9	12
Ohio	65	3	20	12
Missouri	65	3	17	16
Maryland	66	7	19	8
New York	67	6	15	12
District of Columbia	68	5	16	12
Virginia	68	5	11	16
Nevada	68	4	11	16
California	69	11	11	9
Minnesota	69	13	11	8
Colorado	69	8	13	10
Rhode Island	69	6	15	10
Arizona	70	5	12	14
Nebraska	70	6	12	13
Massachusetts	71	6	15	8
Alaska	71	4	12	13
Connecticut	71	6	13	10
New Mexico	71	5	12	13
Kansas	72	2	9	17
Wisconsin	73	6	9	13
Hawaii	76	6	10	8
Delaware	76	2	14	8
Montana	77	0	9	15
Vermont	84	3	3	11

**Table:** 

Age Group, y	Vaccinated With 1 or More Doses, %	Definitely Will Vaccinate, %	Probably Will Vaccinate or Not Sure, %	Probably Will Not or Definitely Will Not Vaccinate, %
18–24	68	9	13	10
25–29	58	7	18	17
30–34	58	6	18	18
35–39	60	5	17	18

By race and ethnicity (Table 2), vaccination coverage was highest among non-Hispanic Asian students (84.4%), followed by Hispanic students (66.9%), non-Hispanic other/multiple races (65.0%), non-Hispanic White (63.8%), and non-Hispanic Black (42.7%). Non-Hispanic Black students (adjusted prevalence ratio [aPR], 0.69; 95% CI, 0.59–0.82), students who sometimes or often did not have enough to eat (aPR, 0.83; 95% CI, 0.70–0.98), and those who found it very difficult to pay for household expenses (aPR, 0.71; 95% CI, 0.62–0.82) were less likely than their counterparts to be vaccinated. Among unvaccinated young adults, those who were non-Hispanic Black (aPR, 1.95; 95% CI, 1.44–2.63), found it very difficult to pay for household expenses (aPR, 1.51; 95% CI, 1.09–2.09), or had a previous diagnosis of COVID-19 (aPR, 1.49; 95% CI, 1.14–1.94) were more likely than their counterparts to report that they probably would get vaccinated or that they were unsure about vaccination (Table 2). Furthermore, adults aged 25 to 29 were more likely than those aged 18 to 24 to report that they did not intend to be vaccinated (aPR, 1.71; 95% CI, 1.21–2.42). Those who often or sometimes did not have enough to eat (aPR, 1.34; 95% CI, 0.98–1.82) and those who found it very difficult to pay for household expenses (aPR, 2.24; 95% CI, 1.53–3.27) were more likely than their counterparts to report that they probably would not or definitely would not get vaccinated.

Among students who would probably get vaccinated or are unsure, the main reasons for not getting vaccinated were the desire to wait and see if it is safe and may get it later (65.3%), concern about possible side effects (59.9%), and the belief that other people need it more right now (35.3%) (Table 3). Among students who reported that they probably will not or definitely will not be vaccinated, reasons for not wanting to be vaccinated were concern about possible side effects (55.0%), lack of trust in COVID-19 vaccines (50.7%), waiting to see if it is safe (41.0%), lack of trust in the government (40.7%), and belief that a vaccine is not needed (39.3%).

**Table 3:** Reasons for Not Getting Vaccinated, by COVID-19 Vaccination Intent, Household Pulse Survey, US, April 14–May 24, 2021^a^

Reason	COVID-19 Vaccination Intent	
	Probably Will/Unsure About Getting the Vaccine, % (95% CI)	Definitely or Probably Will Not Get the Vaccine, % (95% CI)
Plan to wait and see if it is safe and may get it later	65.3 (60.7–69.8)	41.0 (36.6–45.4)
Concerned about possible side effects	59.9 (55.7–64.0)	55.0 (51.1–59.0)
Other people need it more right now	35.3 (30.6–40.0)	16.2 (12.6–19.9)
Don’t trust the government	18.1 (14.6–21.7)	40.7 (36.5–44.9)
Don’t know if a vaccine will work	17.5 (13.4–21.7)	24.4 (20.3–28.4)
Don’t trust COVID-19 vaccines	16.1 (12.6–19.6)	50.7 (46.8–54.6)
Don’t believe I need a vaccine	13.8 (10.3–18.3)	39.3 (34.2–44.7)
Don’t like vaccines	8.1 (5.4–10.8)	16.2 (12.3–20.2)
Concerned about the cost	6.5 (3.3–9.6)	5.1 (2.2–8.0)
Doctor has not recommended it	4.8 (2.9–6.7)	7.5 (4.9–10.1)
Other reason for not getting vaccine	9.3 (7.2–11.4)	14.3 (11.3–17.3)

a All proportions were weighted to be representative of the US population.

## Discussion

We found that nearly 1 in 5 college-aged young adults had been diagnosed with COVID-19, yet 36.9% had not been vaccinated, and 14.0% reported that they probably will not or definitely will not get vaccinated. These findings are concerning, because many college campuses returned to in-person classes in fall 2021, and some colleges do not have vaccination requirements (25). Furthermore, COVID-19 vaccination coverage among US college students varies widely, and some states, particularly those in the South, that have lower coverage and a higher proportion of the population who do not intend to get vaccinated, may increase risk for COVID-19 transmission.

Vaccination coverage among our study population was higher than that found among a nationally representative sample of young adults (15), suggesting that this group may be more responsive to COVID-19 vaccinations than other young adults. However, high vaccination coverage, among all students, is needed for herd immunity. Our study found that college-aged young adults aged 25 or older were less likely than those aged 18 to 24 to get vaccinated, which is in contrast to other studies that found higher vaccination rates among older adults compared with younger adults (14,15). This finding may be due to younger students being more likely to live on campus and get vaccinated because of their close living quarters than older students who may be living off campus or at home. We also found that those who were non-Hispanic Black were least likely among the racial and ethnic groups studied to have been vaccinated and those most likely to have been vaccinated were non-Hispanic Asian, findings that are consistent with other studies (14,15).

The main reasons for not getting vaccinated were waiting to see if the vaccine is safe and concerns about possible side effects. However, among students who probably will not or definitely will not get vaccinated, more than one-half reported that they did not trust COVID-19 vaccines, and about 40% reported that they did not trust the government or believe that they needed a vaccine. This finding is consistent with other studies that found college students to be hesitant toward other vaccines, such as for influenza and human papillomavirus (26,27). Sharing clear and consistent messages about COVID-19 vaccines, increasing confidence in vaccine safety and effectiveness, and highlighting vaccines as important for resuming social activities are important for achieving high vaccination coverage and protecting this population from COVID-19. Furthermore, promoting vaccines on campus and making vaccinations easily accessible to students, such as at student health clinics, may increase vaccination uptake.

Our findings that 43.5% of college-aged young adults reported having anxiety, 39.1% reported depression, 10.9% expressed that they sometimes or often did not have sufficient food, and 34.9% had some or a lot of difficulty covering household expenses highlights the vulnerability of this population and the importance of reducing further adverse health outcomes. Although our study found that college-aged young adults had lower food insufficiency rates than the general population (9), they still experienced vulnerabilities such as anxiety, depression, and financial instability. Vulnerable groups may be less likely to be vaccinated or intend to be vaccinated, and if combined with other comorbidities, this lack of vaccination may increase their risk for serious effects from COVID-19 (28). Some groups, such as non-Hispanic Black adults, may face barriers to vaccination (15). Ensuring high and equitable vaccination coverage among all students is needed to curb the transmission of COVID-19 among students and their families, friends, and communities.

This study has several potential limitations. First, although sampling methods and data weighting were designed to produce nationally representative results, respondents might not be fully representative of the general US adult population (29). Second, the HPS has a low response rate (<10%); however, a nonresponse bias assessment conducted by the US Census Bureau found that the survey weights adjusted for most of this bias, even though some bias may remain (29). Third, vaccination status was self-reported and is subject to social desirability bias. Fourth, although the study attempted to capture college students in this sample, misclassification may have occurred if respondents received some college education and lived with someone else in the household who is currently in college. Fifth, dates of COVID-19 infection and vaccination were not available to assess temporal relationships of these events. Finally, state-level vaccination estimates represent estimates in the state in which college-aged young adults currently live. Because many students may have lived at home during the pandemic, these estimates may not reflect coverage in states in which students go to school.

Vaccination mandates may be useful in increasing vaccination rates among college-aged young adults: previous studies have shown success in improving other childhood vaccination rates (30,31). However, states differ in their legal ability to require COVID-19 vaccinations, the stringency and enforcement of mandates, and the ethical concerns about mandates (32–34). Although an increasing number of colleges are requiring COVID-19 vaccines, many colleges have not implemented mandates. Our study shows that disparities still exist in vaccination coverage and intent among college-aged young adults. In addition to mandates, several studies found that encouraging vaccination for the greater good and making vaccines easily accessible to students may be strong motivators for vaccination (16,17). Emphasizing that COVID-19 vaccines are important for preventing transmission of COVID-19 to family and friends and removing barriers to vaccination are needed to safely reopen in-person learning as well as to resume prepandemic activities.
